# Tissue-Like Phantoms as a Platform for Inserted Fluorescence Nano-Probes

**DOI:** 10.3390/ma9110926

**Published:** 2016-11-15

**Authors:** Tsviya Nayhoz, Eran A. Barnoy, Dror Fixler

**Affiliations:** Faculty of Engineering and the Institute of Nanotechnology and Advanced Materials, Bar Ilan University, Ramat Gan 5290002, Israel; tsviya@gmail.com (T.N.); eabnoy@gmail.com (E.A.B.)

**Keywords:** tissue-like phantoms, dual-modal imaging, fluorescence lifetime (FLT), FLT imaging (FLIM), diffusion reflection

## Abstract

Tissue-like phantoms are widely used as a model for mimicking the optical properties of live tissue. This paper presents the results of a diffusion reflection method and fluorescence lifetime imaging microscopy measurements of fluorescein-conjugated gold nanorods in solution, as well as inserted in solid tissue-imitating phantoms. A lack of consistency between the fluorescence lifetime results of the solutions and the phantoms raises a question about the ability of tissue-like phantoms to maintain the optical properties of inserted contrast agents.

## 1. Introduction

Tissue-like phantoms play a vital role in the development and validation of new imaging technologies. Live tissue features can be replicated by phantoms through control over their optical properties [1,2,3]. Phantoms have been designed to replicate the optical properties of a range of tissues for optical applications such as optical coherence tomography (OCT) [1], magnetic resonance imaging [4], and diffusion reflection (DR) [5]. Such phantoms are a key requirement for the continued development of biomedical techniques and applications. For example: Cubeddu et al. showed the relations, within a phantom, between the concentration of Intralipid (IL) as a scattering component and India ink as an absorbing component to the scattering and absorption coefficients of the phantom, respectively [3]. Early phantoms were based on hydrogels, of which two of the most common were agar [6] and gelatin [7]. Today, other materials are known for the development of versatile tissue-simulating phantoms like: silicone [8], poly(vinyl alcohol) cryogels [9], and fibrin [10]. Phantoms are widely used for a number of purposes including: initial tests of novel systems, routine quality control measurements, performance comparison of different systems, and signal to noise ratio (SNR) optimization in existing systems [1]. This paper presents the initial results of fluorescence lifetime imaging microscopy (FLIM) and DR dual-modal imaging using fluorescein-conjugated-gold nanorods (GNRs), in both solutions and tissue-imitating phantoms. These results, however, raise a question of whether tissue-like phantoms maintain the optical properties of inserted contrast agents.

Biological imaging can be done by a large variety of tools that can image biological tissues. Some of the most frequently used methods are OCT [11], transmission electron microscopy (TEM) [12] and thermal imaging [13]. Biological imaging applications for biomolecular research and diagnostics are endless. One of the challenges in the field of biological imaging is to maximize the information obtained from an image. A way to do it is to combine different methods, a process known as multi-modal imaging. Each imaging method has a set of parameters it extracts, which is different for every method. Moreover, this set of parameters is characterized by factors such as spatial resolution, temporal resolution, detection sensitivity, tissue penetration, SNR, quantitative accuracy, and more [14]. By using more than one imaging technique the advantages of one method can compensate for the drawbacks of the other. This way it is possible to acquire as informative a picture as possible for a specific area [15,16,17].

However, different imaging methods require different contrast agents and it is problematic to simply add two different classes of imaging probes without the same pharmacodynamic properties [18]. Moreover, multiple doses of contrast agents can add stress on the body’s blood clearance mechanism [19]. Therefore, multifunctional integrated contrast agents or probes for multi-modal imaging have been developed to solve this problem. Various methods have been applied to achieve multimodal functionality in a single probe unit. Some of them are: lipid-based approaches that use lipid carrier systems as vehicles [20,21,22,23,24], macromolecular carriers that form multifunctional probes by coupling different types of contrast agents to a common macromolecule [19,25,26], small molecule multimodal probes [19], and organic and inorganic nanoparticles that are fabricated or modified into multifunctional probes by conjugation to molecules, load encapsulation using a core and/or shell, or doping with various materials [19].

A dual-modal imaging technique that combines FLIM and DR using fluorophore-conjugated gold nano-particles (GNPs) has been presented recently by our research group [27,28]. However, the aspect of the separation distance between the GNPs and fluorophores had not been explored in such easy-to-manufacture and simple probes. Metallic nanostructures have a strong interaction with incident light, which results in the generation of surface plasmons (SPs) in the metal. Excited fluorophores (as dipoles) within a short distance of the metal (less than 50 nm) interact strongly with those SPs [29]. This interaction results in FLT shortening and increased (metal enhanced fluorescence) [30,31] or decreased [32] quantum yield. The interaction is distance and dipole orientation dependent [33,34,35]. In the experiment presented here, fluorescein-conjugated-GNRs with different conjugation distances have been fabricated and examined to optimize the nano-probes. DR measurements of fluorescein-conjugated-GNR phantoms were performed, as well as time-domain FLIM measurements of fluorescein-conjugated-GNRs, both in solution and inserted in tissue-like phantoms. Although the DR measurements behaved as hypothesized, the FLIM results revealed that there was no consistent relationship between the FLT measurements of the solutions and the phantoms with the same nano-probes. This discrepancy leads us to assume that the tissue-like phantoms do not maintain the optical properties of their inserted contrast agents.

## 2. Results

In order to examine the DR system’s ability to detect the fluorescein-conjugated-GNR nano-probes and their concentration, 12 solid phantoms that contain fluorescein-conjugated-GNRs were made as described in the *Materials and Methods* section: six with conjugation through 11-amino-1-undecanethiol linker and six with conjugation through 6-amino-1-hexanethiol linker. The Au concentration of each set of six phantoms varied from 0.05 mg/mL to 0.3 mg/mL by steps of 0.05 mg/mL. In addition, control phantoms containing fluorescein with matching fluorescein concentrations were made as well.

**Figure 1:**
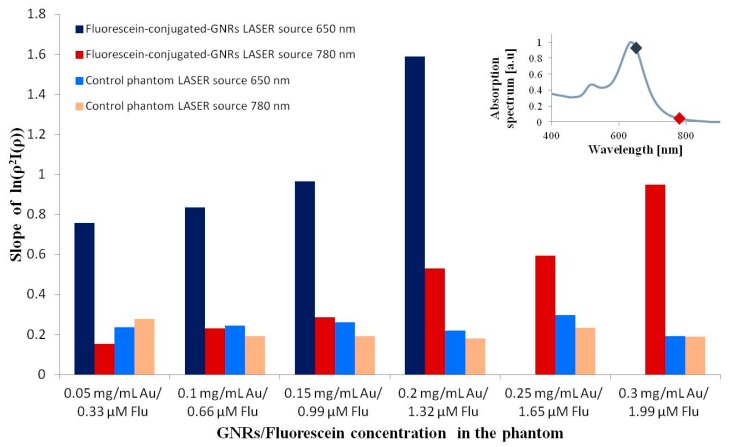
A bar plot of ln(ρ^2^Γ(ρ)) slopes of the different phantoms, which were measured using the DR method. In the upper right corner, the absorption spectrum of the GNRs is presented with the DR light source wavelengths marked by diamonds.

DR measurements of the phantoms described above were performed. The reflected light intensity from the different solid phantoms was measured using a DR set-up with laser sources of 650 and 780 nm as described in the *Materials and Methods* section. The slope of ln(ρ^2^Γ(ρ)) was calculated, where Γ(ρ) describes the reflected light intensity at the phantom surface in several light source-detector separations (represented by ρ). Figure 1 shows a bar plot of the ln(ρ^2^Γ(ρ)) slopes of the fluorescein-conjugated-GNR phantoms with different GNR concentrations and the control phantom with matching dye concentrations.

Solutions and tissue-like phantoms with fluorescein, each with a fluorescein concentration of 0.33 µM, were made. Fluorescein was conjugated to GNRs using linkers of varying lengths, and also prepared in solutions and phantoms. These solutions had a fluorescein concentration of 6.6 µM, and the phantoms had a concentration of 0.33 µM. All samples were measured using the time-domain FLIM system. The system’s excitation rate was 50 MHz, the pinhole diameter 2 mm and the sample was scanned to an image with a varied number of pixels up to a maximum of 64 × 64 pixels.

**Figure 2:**
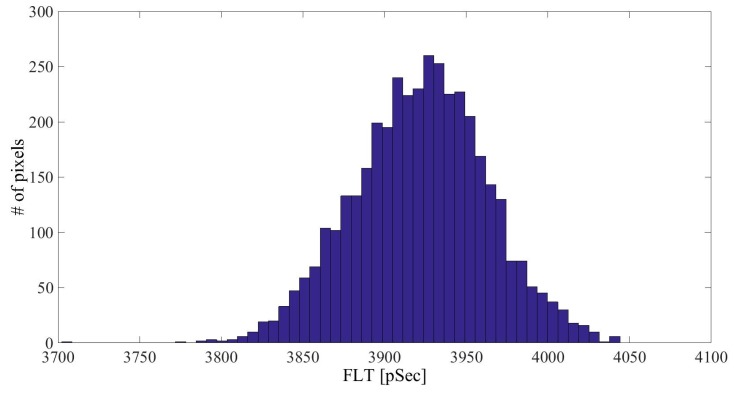
FLT histogram of fluorescein 0.33 µM solution.

**Figure 3:**
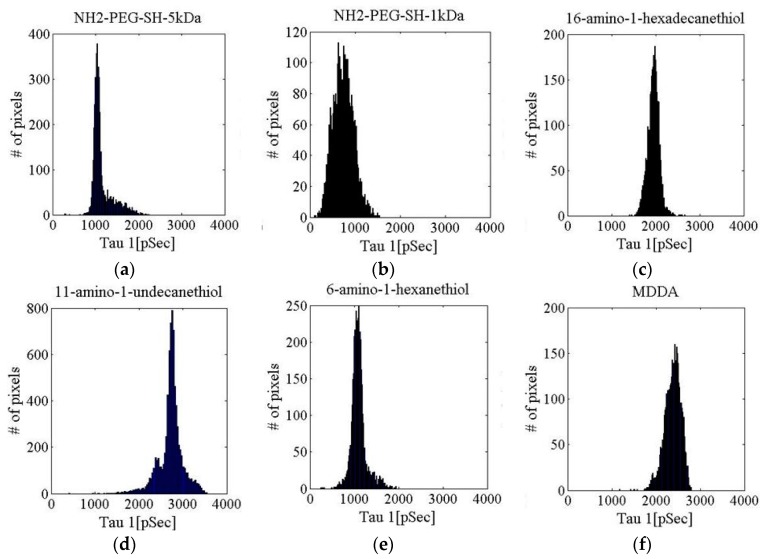
τ_1_ histograms for fluorescein-conjugated GNR solutions with different linkers: (**a**) NH_2_-PEG-SH-5 kDa; (**b**) NH_2_-PEG-SH-1 kDa; (**c**) 16-amino-1-hexadecanethiol; (**d**) 11-amino-1-undecanethiol; (**e**) 6-amino-1-hexanethiol; (**f**) MDDA (Au concentration 1 mg/mL, fluorescein concentration 6.6 µM).

The fluorescence intensity (FI) decay of each pixel of the fluorescein solution sample was fitted to a mono-exponential curve. An average over all of the pixels yielded a FLT of 3.92 ± 0.04 ns (Figure 2 shows a histogram of τ_1_ for the fluorescein solution measurement). The FI decay of each pixel of the fluorescein-conjugated-GNR solutions was fitted to a bi-exponential function where τ_2_ was fixed to 3.92 ns (the FLT measured for free fluorescein solution). The average values and STD of τ_1_, a_1_% (the percentage of τ_1_ of the pixel’s FLT), and χ^2^ (the fit quality parameter), were calculated and are summarized in Table 1, and Figure 3 presents the τ_1_ histograms for the fluorescein-conjugated-GNR solutions.

**Figure 4:**
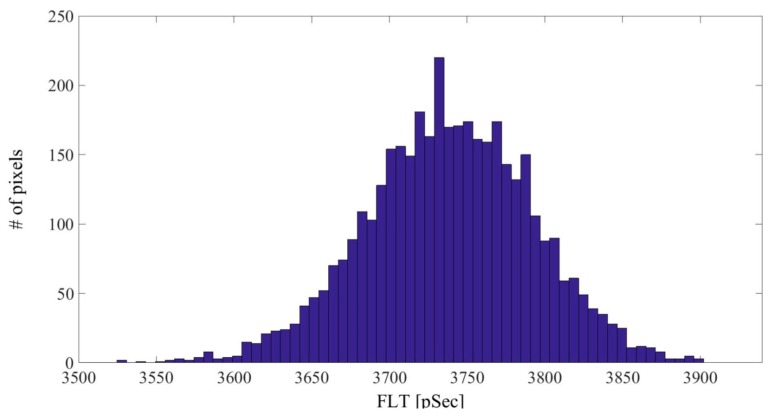
FLT histogram of free fluorescein phantom. The fluorescein concentration in the phantom was 0.33 μM.

**Figure 5:**
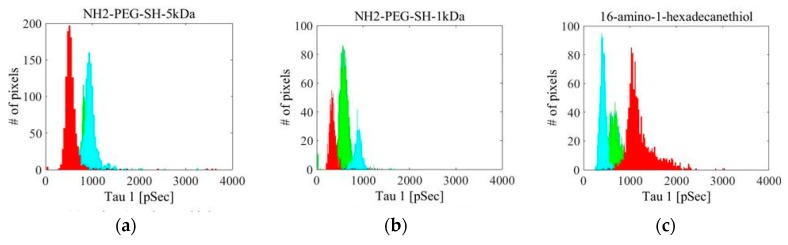

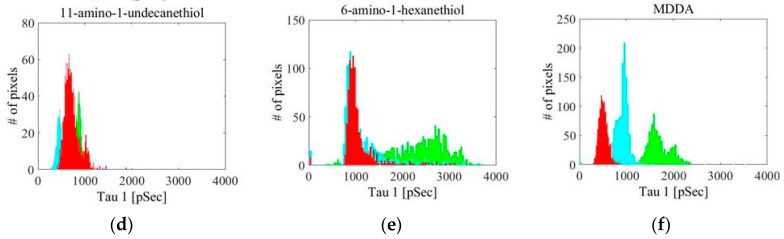
τ_1_ histograms for fluorescein-conjugated GNR phantoms with different linkers: (**a**) NH_2_-PEG-SH-5 kDa; (**b**) NH_2_-PEG-SH-1 kDa; (**c**) 16-amino-1-hexadecanethiol; (**d**) 11-amino-1-undecanethiol; (**e**) 6-amino-1-hexanethiol; (**f**) MDDA. Histograms are shown for three areas indicated by the different colors. In all phantoms, Au concentration was 0.05 mg/mL, and fluorescein concentration was 0.33 µM.

The FI decay of each pixel of the free fluorescein phantom was fit to a mono-exponential curve. An average over all of the pixels yielded a FLT of 3.74 ± 0.06 ns. Figure 4 shows a histogram of τ_1_ for the fluorescein phantom measurement. The fluorescein-conjugated-GNR phantoms were measured at three different areas. The FI decay of each pixel of the fluorescein-conjugated-GNR phantom images was fit to a bi-exponential function where τ_2_ was fixed to 3.74 ns (the FLT measured for the free fluorescein phantom). The average values and STD of τ_1_, a_1_% (the percentage of τ_1_ of the pixel’s FLT), and χ^2^ (the fit quality parameter), were calculated and are summarized in Table 2, and the corresponding τ_1_ histograms are presented in Figure 5.

## 3. Discussion and Conclusions

The results described above establish that fluorescein-conjugated-GNRs are good multi-functional nano-probes for FLIM and DR dual-modal imaging. The DR results (see Figure 1) show a high correlation between the ln(ρ^2^Γ(ρ)) slopes and the GNR concentration in the phantom: the higher the GNR concentration, the greater the slope, which indicates more intense absorption. Moreover, steeper slopes are observed for the 650 nm light source due to the GNRs’ higher absorption in this wavelength. It should be noted that, for the 650 nm light source, the DR was not able to provide values if the GNR concentration was too high. This happens because, in high concentration, the absorption is dominant and all of the light is absorbed for particles of this geometry. In a similar manner, despite the absorption spectrum suggesting a low absorption at 780 nm, the correlated increase in slope with GNR concentrations is apparent even for higher concentrations with this light source. In addition, a clear difference is observed between the control phantoms that contain only free fluorescein and the corresponding fluorescein-conjugated-GNR phantoms.

**Figure 6:**
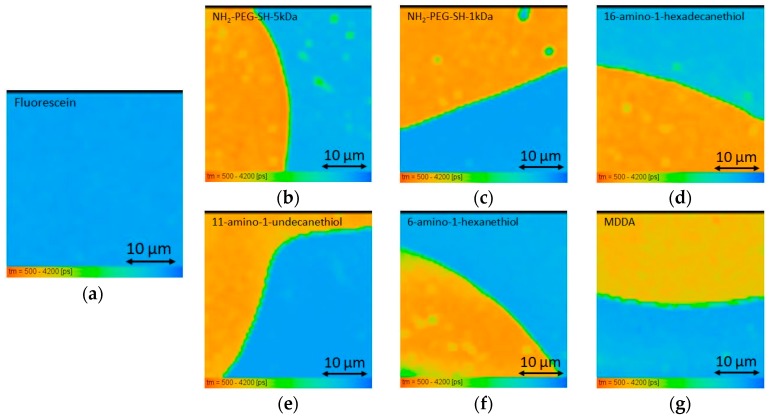
FLIM images of (**a**) free fluorescein control phantom, and the image of the interface region of base phantoms containing free fluorescein (fluorescein concentration of 330 nM) and phantoms containing fluorescein-conjugated-GNRs with conjugation through different likers: (**b**) NH_2_-PEG-SH-5 kDa; (**c**) NH_2_-PEG-SH-1 kDa; (**d**) 16-amino-1-hexadecanethiol; (**e**) 11-amino-1-undecanethiol; (**f**) 6-amino-1-hexanethiol; (**g**) MDDA (Au concentration 0.05 mg/mL, fluorescein concentration of 330 nM). Color indicates the average FLT in each pixel.

**Figure 7:**
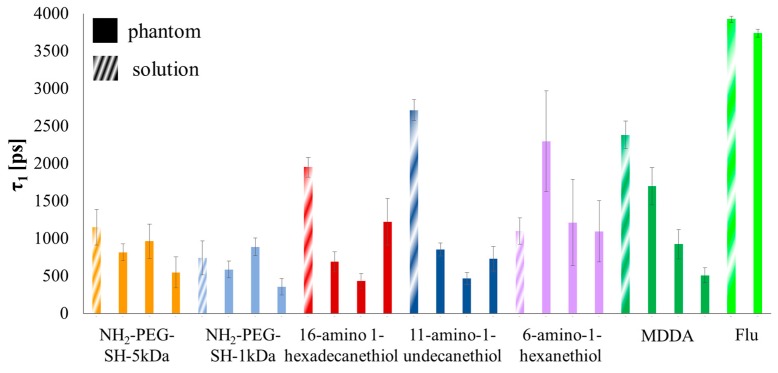
Bar plot of τ_1_ for fluorescein-conjugated GNRs with different linkers’ solutions and phantoms (indicated by striped or solid texture respectively), where three area measurements are shown for each phantom. Free fluorescein solution and phantom FLTs are shown at the end for comparison.

The FLIM results described above clearly show that the conjugation of fluorescein to GNRs shortens its FLT significantly for all the conjugations that were examined in this experiment, both in solution and inserted in solid tissue-imitating phantoms (Figure 6 and Figure 7). This result is consistent with known fluorophore interactions with a nearby metal [33,35,36]. FLIM and DR dual-modal imaging using fluorescein-conjugated GNRs was achieved.

However, the observed FLT shortening showed no correlation to the linker length. The near-field effects of metallic nanoparticles should gain strength with decreasing distance, meaning that as a fluorophore gets closer to a particle it should exhibit a shorter FLT [30,33]. In the current experiment, the linkers separating the fluorescein and GNRs were chosen to vary in length from around 1 nm up to about 50 nm, but the FLT values, in both solution and phantoms, did not necessarily increase with the linker length. This problem might have occurred because the chosen linkers might not be rigid enough to maintain their full length at all times.

In addition, when comparing the solution and phantom measurements, the results indicate a potential serious problem with the use of phantoms. The conjugation via the 16-amino-1-hexadecanethion (estimated length 2.5 nm) and 11-amino-1-undecanethion (estimated length 1.7 nm) linkers show a large discrepancy between the FLT measurements of the fluorescein-conjugated-GNRs in solution and in phantoms. This difference is also noted in the 6-amino-1-hexanthiol (estimated length 0.9 nm) and MDDA to a lesser extent. Meanwhile, for the NH_2_-PEG-SH-5 kDa and NH_2_-PEG-SH-1 kDa (estimated length 50 nm and 10 nm, respectively), the change from solution to phantom is much less pronounced (see Figure 3, Figure 5 and Figure 6). It seems that the phantoms affect the probes and change their properties to extents that vary based on the linkers separating the GNRs and fluorophores, though not necessarily in correlation with the end-to-end lengths of the linkers. It is possible that in a phantom, a linker’s configuration changes due to the viscosity created by the solidification of the phantom, and subsequently, differences are detected between phantoms and solutions. The NH_2_-PEG-SH-5 kDa and NH_2_-PEG-SH-1 kDa linkers are significantly longer molecules than the others so that they might fold upon themselves both in solutions and in phantoms and, therefore, the effect of their insertion into phantoms is minimized.

Moreover, there is no clear consistency in the FLT results of different areas within the same phantoms (see Figure 5 and Figure 6). This incongruity, as well as the observed difference in the FLT measurements of free fluorescein in solution and phantom (see Figure 6), leads us to assume that the solidification process of the phantoms might also affect the spatial distribution of the nano-probes, creating areas of higher and lower concentrations, which might affect the phantom results as well.

In conclusion, fluorescein-conjugated-GNRs placed in tissue-imitating phantoms pose as viable dual-imaging probes for DR and FLIM, albeit with caveats. One issue arises from the use of non-rigid linkers, which will not necessarily retain their spatial configuration. Another issue is that it is not clear that tissue-like phantoms can maintain the optical properties of contrast agents inserted into them and, therefore, the usage of phantoms as a model for measuring the behavior of inserted probes needs to be carefully examined for each probe of interest.

## 4. Materials and Methods

### 4.1. GNRs Fabriction

**Figure 8:**
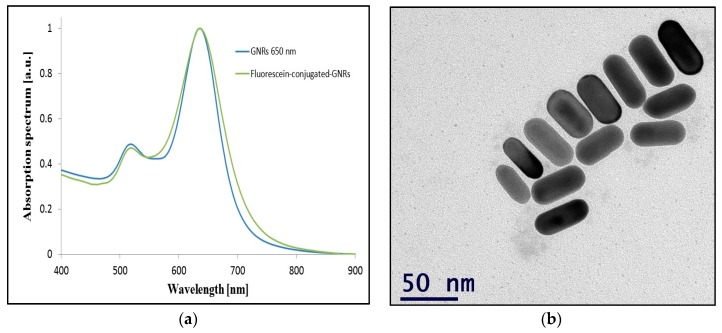
GNRs characteristics: (**a**) GNRs normalized absorption spectrum; (**b**) GNRs TEM image.

GNRs with an absorption peak of approximately 650 nm were synthesized using the seed-mediated growth method [37]. The absorption spectrum of the GNRs was verified by a spectrophotometer before and after conjugation to fluorescein (see Figure 8a), after conjugation to fluorescein the absorption spectrum becomes broader due to slight changes in the particles’ geometry and the addition of another refractive index indicating the conjugation as expected. The GNRs’ shape and size were verified by transmission electron microscopy (TEM) (see Figure 8b).

### 4.2. Flourescein Conjugation to GNRs

**Figure 9:**
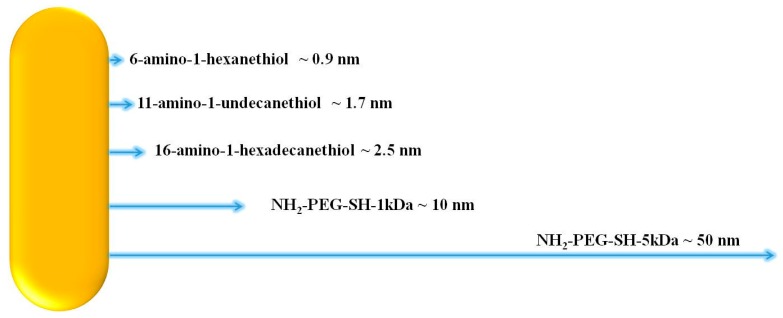
A schematic representation of the linkers’ end-to-end length in scale relatively to each other.

**Figure 10:**
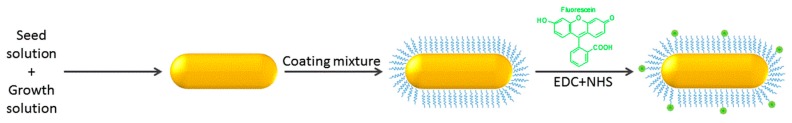
A schematic representation of the GNRs’ fabrication and fluorescein conjugation process.

GNRs were coated with a linker mixture (the different mixtures are summarized in Table 3 and a schematic representation of their varying length is presented in Figure 9) by adding the linker mixture to the GNR solution and stirring for at least 2 h, after which the fluorescein solution was added to create either covalent or overlap binding with the linkers. In cases of covalent binding, EDC (1-Ethyl-3-(3-dimethylaminopropyl) carbodiimide HCl) and NHS (*N*-Hydroxysulfosuccinimide sodium salt), which are activating agents that help to form the desirable bond by creating good leaving groups, were added as well [38]. Similar binding has been done before [39]. The GNR solutions were then left to stir overnight. A schematic representation of the process is shown in Figure 10. In order to wash unattached fluorescein, the solutions were diluted with DDW and centrifuged until precipitation of the GNRs, and a clear suspension was obtained. The MDDA linker does not bind to the GNRs through a SH group but rather envelops the GNRs.

### 4.3. Solid Tissue-Like Phantom Preparation

For the DR measurements, 12 solid phantoms were prepared with fluorescein-conjugated-GNRs: six with conjugation through 11-amino-1-undecanethiol linker and six with conjugation through 6-amino-1-hexanethiol linker. The Au concentration of each set of six phantoms varied from 0.05 to 0.3 mg/mL by steps of 0.05 mg/mL, and the phantoms had a final volume of 400 µL. In addition, control phantoms were prepared containing fluorescein with matching fluorescein concentrations (0.33 to 1.98 µM by steps of 0.33 µM—dye concentrations matching the Au concentrations) and final volume of 4 mL.

For the FLIM measurements, a total of seven solid phantoms were prepared: one free fluorescein phantom with a total volume of 4 mL and final fluorescein concentration of 0.33 µM, and six fluorescein-conjugated-GNR phantoms with a total volume of 400 µL and final Au concentration of 0.05 mg/mL.

The phantoms were prepared by mixing Intra Lipid (IL) (Lipofundin MCT/LCT 20%, B. Braun Melsungen AG, Melsungen, Germany) as a scattering component, India ink (ink solution diluted to 0.1% ink) as an absorption component, the contrast solution of choice (free fluorescein solutions or fluorescein-conjugated-GNR solutions), double distilled water (DDW), and agarose powder (LONZA SeaKem^®^ LE Agarose, Walkersville, MD, USA) for solidification into a gel. The materials’ ratios in the final total volume are specified in Table 4. For example, the free fluorescein FLIM phantom was made of: 400 µL Intralipid (IL), 120 µL India ink, 132 µL fluorescein solution (10 µM), 3.348 mL DDW, and 40 mg agarose powder. Each of the fluorescein-conjugated-GNR FLIM phantoms were made of: 40 µL IL, 12 µL India ink, 20 µL fluorescein-conjugated-GNRs solution (Au concentration 1 mg/mL), 328 µL DDW, and 4 mg agarose powder.

The phantoms were prepared as follows: first, all of the ingredients but the agarose were added into a glass vial. The solution was heated and stirred. When the solution was hot agarose powder was added slowly. Once the solution was well mixed it was transferred into 12-well or 96-well tissue culture plates and cooled under vacuum (to avoid bubbles).

### 4.4. DR Measurments

**Figure 11:**
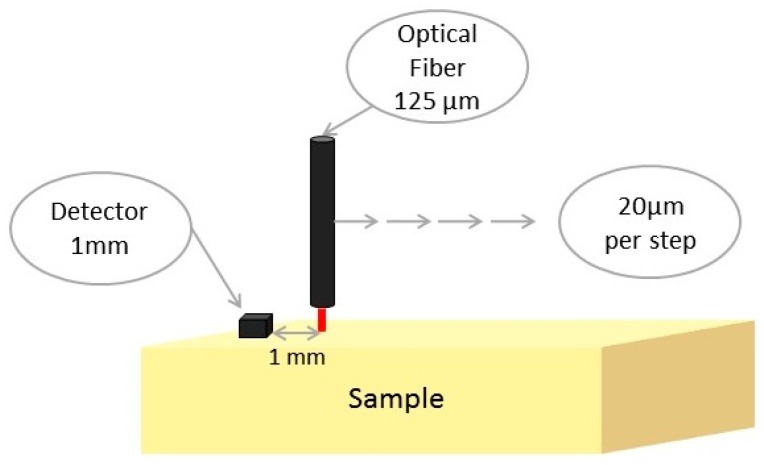
A schematic representation of the DR system.

A DR system was designed and built (NEGOH-OP TECHNOLOGIES, Israel), as was previously described [40]. The setup includes two laser diodes with wavelengths of 650 and 780 nm as excitation sources. Irradiation is carried out using a 125 μm diameter optic fiber to achieve a pencil beam illumination. A portable photodiode is used as a photo detector (a schematic of the setup is shown in Figure 11) The expected reflected light intensity as a function of the distance between the light source and the detector profile, Γ(ρ), is defined by:

(1) $$\Gamma \left(\rho \right)=\frac{c_{1}}{\rho^{2}}\exp\left(-µ\rho \right),$$

where $µ=\sqrt{3{µ}_{a}{µ}_{s}\prime }$ and is the effective attenuation coefficient, µ_a_—the absorption coefficient, µ_s_’—the reduced scattering coefficient, and ρ—the source-detector separation. This equation can be rewritten as:

(2) $$\ln\left(\rho^{2}\Gamma \left(\rho \right)\right)=c_{2}-µ\rho ,$$

where *c*_2_ is ln(*c*_1_). From these equations the values of the absorption and reduced scattering coefficients can be extracted.

### 4.5. FLIM Measurments

**Figure 12:**
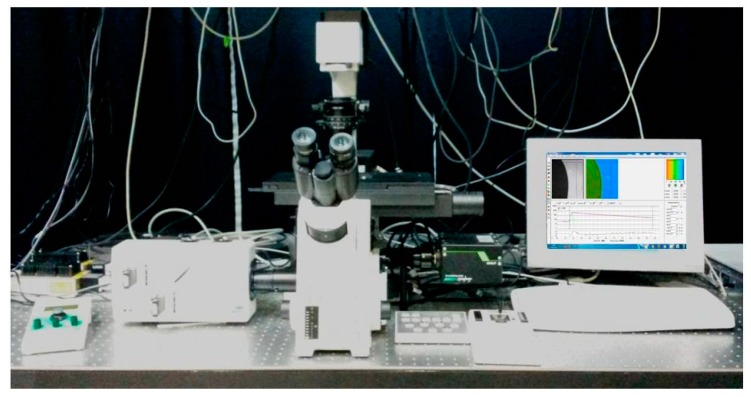
An image of the FLIM system.

The FLIM system (see in Figure 12) used for FLT measurements in this article is a two-channel laser scanning confocal microscope (DCS 120, Becker and Hickl GmbH, Berlin, Germany) with two FLIM detectors. The system excites every pixel of the sample with laser pulses, at a frequency of 20, 50, or 80 MHz. The FWHM of the excitation pulse is of the order of 10 ps–100 ps and the excitation wavelength is 470 nm. The excitation creates fluorescence, and the system detects the FI using a time correlated single photon counting (TCSPC) card. FLTs are typically 0.1 ns–10 ns. The fluorescence decay is fit to an exponential or sum of exponentials model by:

(3) $$I\left(t\right)=\sum \;\alpha_{i}\exp\left(-\frac{t}{\tau_{i}}\right),$$

The amplitude-weighted FLT is calculated as defined:

(4) $$<\tau >=\sum \;\alpha_{i}\tau_{i},$$

## Figures and Tables

**Table 1:** This table summarizes the results of τ_1_, a_1_% (the percentage of τ_1_ of the pixel’s FLT), χ^2^ (the fit quality parameter) average, and the standard deviation (STD) values for fluorescein-conjugated GNRs with different linkers.

Linker Type	Estimated Linker Length [nm]	a_1_%—Average	a_1_%—STD	τ_1_—Average [ns]	τ_1_—STD [ns]	χ^2^—Average	χ^2^—STD
NH2-PEG-SH-5 kDa	50	81.16	6.13	1.15	0.24	1.312	0.535
NH2-PEG-SH-1 kDa	10	91.15	6.52	0.74	0.23	1.461	0.327
16-amino-1-hexadecanethiol	2.5	87.02	3.55	1.95	0.13	1.609	0.149
11-amino-1-undecanethiol	1.7	66.13	13.49	2.72	0.28	1.366	0.332
6-amino-1-hexanethiol	0.9	81.03	6.46	1.10	0.17	1.259	0.360
3,4-methylenedioxy-*N*,*N*-dimethylamphetamine (MDDA)	Unkown	80.84	6.84	2.38	0.18	1.124	0.132

**Table 2:** This table summarizes the results of τ_1_, a_1_% (the percentage of τ_1_ of the pixel’s FLT), χ^2^ (the fit quality parameter average), and standard deviation (STD) values for fluorescein-conjugated GNRs with different linkers. Each phantom was measured in three areas, marked as (1), (2), and (3) for each phantom. In all phantoms, Au concentration was 0.05 mg/mL and fluorescein concentration was 0.33 µM.

Linker Type	a_1_%—Average	a_1_%—STD	τ_1_—Average [ns]	τ_1_—STD [ns]	χ^2^—Average	χ^2^—STD
NH2-PEG-SH-5 kDa (1)	81.31	5.00	0.81	0.11	5.25	1.02
NH2-PEG-SH-5 kDa (2)	78.18	7.57	0.96	0.23	2.86	0.53
NH2-PEG-SH-5 kDa (3)	70.10	10.31	0.54	0.21	2.71	0.55
NH2-PEG-SH-1 kDa (1)	65.29	8.31	0.58	0.11	1.25	0.13
NH2-PEG-SH-1 kDa (2)	81.77	3.72	0.89	0.11	2.75	0.37
NH2-PEG-SH-1 kDa (3)	74.38	3.33	0.35	0.11	1.67	0.21
16-amino-1-hexadecanethiol (1)	78.15	3.84	0.69	0.13	3.74	0.70
16-amino-1-hexadecanethiol (2)	72.23	4.48	0.43	0.10	2.12	0.34
16-amino-1-hexadecanethiol (3)	77.46	8.68	1.22	0.31	2.01	0.36
11-amino-1-undecanethiol (1)	79.80	3.71	0.85	0.08	7.29	1.37
11-amino-1-undecanethiol (2)	65.91	3.63	0.46	0.08	3.38	0.60
11-amino-1-undecanethiol (3)	67.60	6.82	0.73	0.16	4.40	0.87
6-amino-1-hexanethiol (1)	50.15	18.90	2.29	0.67	1.19	0.18
6-amino-1-hexanethiol (2)	50.99	13.16	1.21	0.57	1.60	0.65
6-amino-1-hexanethiol (3)	52.15	10.57	1.09	0.41	1.35	0.79
MDDA (1)	82.41	9.83	1.69	0.25	1.20	0.16
MDDA (2)	74.63	9.08	0.92	0.19	3.90	0.79
MDDA (3)	60.76	3.29	0.51	0.10	1.81	0.24

**Table 3:** This table lists the different linkers’ mixtures, the percentage of each linker in these mixtures and the matching fluorescein coating percentage.

Linker A	% Coating A	Estimated Linker Length [nm]	Linker B	% Coating B	% Coating Fluorescein	Binding Type
NH2-PEG-SH-5 kDa (MW 5000 g/mol)	10%	50	mPEG-SH-5 kDa (MW 5000 g/mol)	90%	10%	Covalent binding
NH2-PEG-SH-1 kDa (MW 5000 g/mol)	10%	10	mPEG-SH-1 kDa (MW 1000 g/mol)	90%	10%	Covalent binding
16-amino-1-hexadecanethiol (MW 309.98 g/mol)	10%	2.5	1-octanethiol 98.5+% (MW 146.29 g/mol)	90%	10%	Covalent binding
11-amino-1-undecanethiol (MW 239.85 g/mol)	10%	1.7	1-octanethiol 98.5+% (MW 146.29 g/mol)	90%	10%	Covalent binding
6-amino-1-hexanethiol (MW 169.72 g/mol)	10%	0.9	1-octanethiol 98.5+% (MW 146.29 g/mol)	90%	10%	Covalent binding
3,4-methylenedioxy-*N*,*N*-dimethylamphetamine (MDDA) (MW 207.26888 g/mol)	100%	-	-	-	10%	Overlap binding

**Table 4:** Specification of the volume’s ratios of the materials needed for a phantom preparation.

IL	India Ink (Diluted to 0.1%)	Wanted Solution	DDW	Agarose Powder
10% of the total volume	3% of the total volume	X% of the total volume (depends on the wanted concentration in the phantom)	(87−X)% of the total volume	1% (defined as 1 g per 100 mL)
